# Integrated Transcriptomic Analyses Identify Four Prognosis-Associated Genes in Hepatocellular Carcinoma

**DOI:** 10.3390/ijms27177535

**Published:** 2026-08-23

**Authors:** Yuxian Liu, Xingjie Chen, Junyuan Zhang, Xueyan Zhou, Xiaohui Li, Kangcheng Xu, Hao Lin, Yanni Cao

**Affiliations:** 1 School of Artificial Intelligence, Anhui University of Science & Technology, Huainan 232001, China; yxl@aust.edu.cn (Y.L.); chenxj340@163.com (X.C.); 2024201872@aust.edu.cn (J.Z.); xueyan_zz@163.com (X.Z.); 2023201661@aust.edu.cn (X.L.); 2024201843@aust.edu.cn (K.X.); 2 State Key Laboratory of Digital Intelligent Technology for Unmanned Coal Mining, Anhui University of Science & Technology, Huainan 232001, China; 3 Key Laboratory for Neuro-Information of Ministry of Education, Center for Informational Biology, School of Life Sciences and Technology, University of Electronic Science and Technology of China, Chengdu 611731, China

**Keywords:** machine learning, prognosis, gene expression, hepatocellular carcinoma, clinical, immune microenvironment, drug

## Abstract

Hepatocellular carcinoma (HCC) is one of the malignant tumors with high incidence and mortality rates worldwide. Given the poor prognosis of patients with HCC, it is crucial to explore the molecular mechanisms underlying HCC development and to evaluate prognostic markers. Differential expression analysis followed by univariate Cox, LASSO, and multivariate Cox regression identified four genes (EPO, SOCS2, IL18RAP, and KPNA2), and a Cox-based risk score was evaluated in the TCGA-LIHC cohort and externally in GSE14520 using Kaplan–Meier and time-dependent ROC analyses. Bulk, single-cell, and protein resources provided convergent expression context. Survival machine-learning analysis using observed overall-survival time and censoring status identified Cox–Ridge as the best-performing model in TCGA-LIHC, with more modest performance in GSE14520, and immune profiling revealed risk-group-associated differences in estimated immune and stromal components, immune-cell composition, and immune-checkpoint expression. The oncoPredict/GDSC2 screen highlighted five potential drug candidates for experimental prioritization. Because the drug screen is based on computationally predicted sensitivities, these findings should be regarded as hypothesis-generating and require validation in prospective cohorts and experimental systems before clinical translation.

## 1. Introduction

Hepatocellular carcinoma (HCC) is acknowledged as the most frequent malignant tumor originating from liver tissues, ranking sixth in global disease burden and third in cancer deaths [1,2]. For 2026, the projected burden of HCC in the United States is 42,340 new diagnoses and 30,980 new deaths attributed to the disease [3]. Worldwide estimates further indicate a marked increase by 2040, when HCC may account for about 1.4 million newly diagnosed patients and 1.3 million deaths [4]. Great progress has been achieved in early detection, potential biomarker identification, and treatment regimens for HCC in recent years [5,6]. However, the prognosis and survival of patients remain pessimistic. Prognostic studies of HCC patients not only help to reveal the underlying molecular mechanisms, but also provide important evidence for precision medicine. Therefore, the prognostic analysis of HCC has become a key research focus [7].

Owing to the rapid development of high-throughput sequencing, machine learning (ML) algorithms [8,9,10,11,12,13,14], and bioinformatics analysis methods in recent years, researchers have been able to systematically reveal the molecular characteristics of HCC at the transcriptomic level [15,16,17,18,19,20,21]. By analyzing gene expression levels and regulatory networks, transcriptome studies provide vital clues for understanding the progression and pathogenic mechanisms of HCC, thereby guiding the exploration of biomarkers for early diagnosis of HCC [22,23,24]. Using single-cell RNA sequencing data, Cui et al. have constructed a prognosis-oriented model to assess survival risk in HCC patients [25,26]. In another study, 12 gene signatures have been identified and incorporated into a risk model for predicting 1-, 3-, and 5-year OS, producing AUCs of 0.77, 0.73, and 0.72 [27]. Additionally, it has been reported that ML-based prognostic models can improve the reliability of OS predictions for patients [28]. For example, Kang et al. have constructed survival prediction models of breast cancer patients using extra survival trees, gradient boosting survival, penalized Cox algorithms (least absolute shrinkage and selection operator (LASSO) and ElasticNet), and random survival forest, respectively. Among them, the extra survival trees model (AUC=0.83 in the older groups and AUC=0.87 in the younger groups) outperforms other ML models in predicting survival [29]. Dong et al. have used six ML algorithms (k-nearest neighbor (KNN), logistic regression (LR), support vector machine (SVM) [30], extreme gradient boosting (XGBoost), random forest (RF) [31], and decision tree (DT) [32]) to predict the survival status of alpha-fetoprotein-positive HCC patients. Among all algorithms, XGBoost exhibits the optimal predictive capability. In the external validation cohort, its AUC values regarding 1-, 3-, and 5-year OS are calculated as 0.782, 0.749, and 0.740 [33]. ML algorithms, including extremely randomized trees (ET), RF, and XGBoost, have also been applied to survival prediction in other cancer types [34]. Although numerous transcriptome-derived and machine-learning-based prognostic signatures have been proposed for HCC, their reported performance is difficult to compare directly because the models were developed in different cohorts and used different gene compositions, expression platforms, survival endpoints, preprocessing procedures, and validation strategies [27,33,35,36]. A compact gene signature may offer greater interpretability and facilitate practical cross-cohort evaluation, but its prognostic utility still requires assessment in independent datasets. Despite these advances, the reproducibility and practical interpretability of HCC prognostic signatures remain variable across cohorts. Regarding key biomarkers that play a regulatory role in HCC, studies have revealed that upregulation of GULP1 can promote the growth of HCC cells [37]. Silencing of CD2AP inhibits the proliferation of HCC cells [38]. Although several regulatory mechanisms have been reported, the molecular complexity of HCC remains incompletely understood. Accordingly, integrating transcriptomic analysis with transparent ML evaluation may help prioritize prognosis-associated candidates and generate hypotheses for subsequent biological and therapeutic investigation.

In this paper, we investigated transcriptional patterns in HCC and normal tissues and evaluated whether a compact four-gene Cox signature could stratify patient outcomes across independent cohorts. We further examined the expression context of these genes using bulk transcriptomic, single-cell, and protein-level resources. Survival machine-learning analyses, immune microenvironment analyses, and drug sensitivity analyses were conducted to generate testable biological and therapeutic hypotheses.

## 2. Results

### 2.1. Differences in Gene Expression Between HCC and Normal Tissues

**Figure 1:**
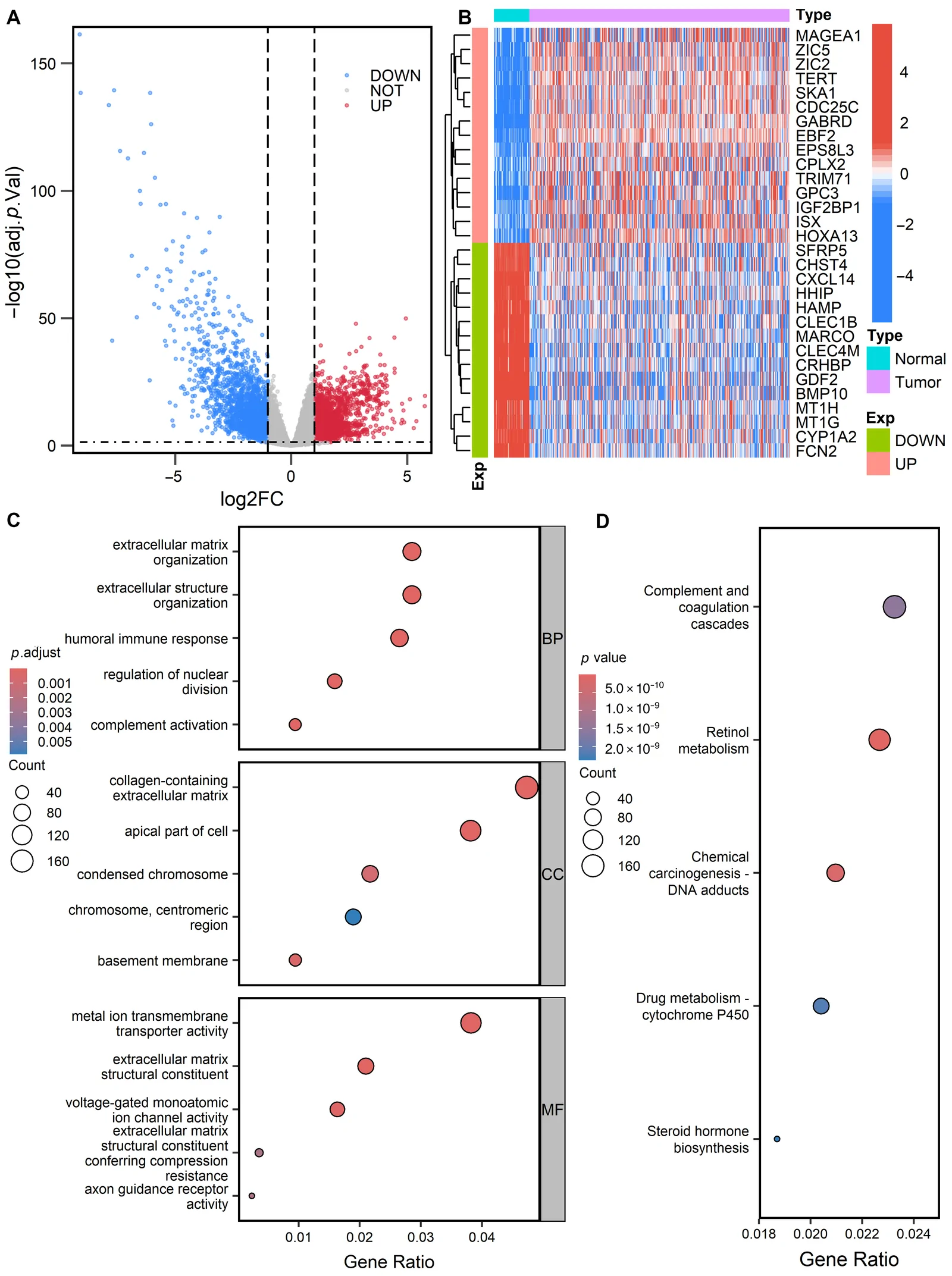
DEGs between HCC and normal tissues. (**A**) Volcano plot of DEGs. The two vertical dashed lines in the figure represent the screening thresholds for DEGs ($\left|\log_{2}FoldChange\right|=1$). The horizontal dashed line corresponds to the screening threshold for statistical significance (Benjamini–Hochberg adjusted p=0.05). (**B**) The expression of the top 15 DEGs in two tissues. (**C**) The GO functional enrichment analysis of DEGs. (**D**) The KEGG pathway of DEGs.

To assess transcriptional differences between HCC and normal tissues, we analyzed TCGA-LIHC samples and identified 1783 upregulated and 2038 downregulated genes (Figure 1A). The 15 selected DEGs showed clear differential expression between HCC and normal samples (Figure 1B). GO and KEGG enrichment analyses were then performed for the 3821 DEGs. GO biological-process terms included extracellular structure organization, extracellular matrix organization, and humoral immune response. GO cellular-component terms included collagen-containing extracellular matrix, condensed chromosome, and apical part of cell. In contrast, molecular function terms included extracellular matrix structural constituent and metal ion transmembrane transporter activity (Figure 1C). KEGG analysis highlighted complement and coagulation cascades, chemical carcinogenesis-DNA adducts, and retinol metabolism (Figure 1D). These enrichment results provided transcriptional context for the identified DEGs and a basis for subsequent prognostic analyses.

### 2.2. Construction and Validation of the Prognostic Risk Scoring Model for HCC Patients

**Figure 2:**
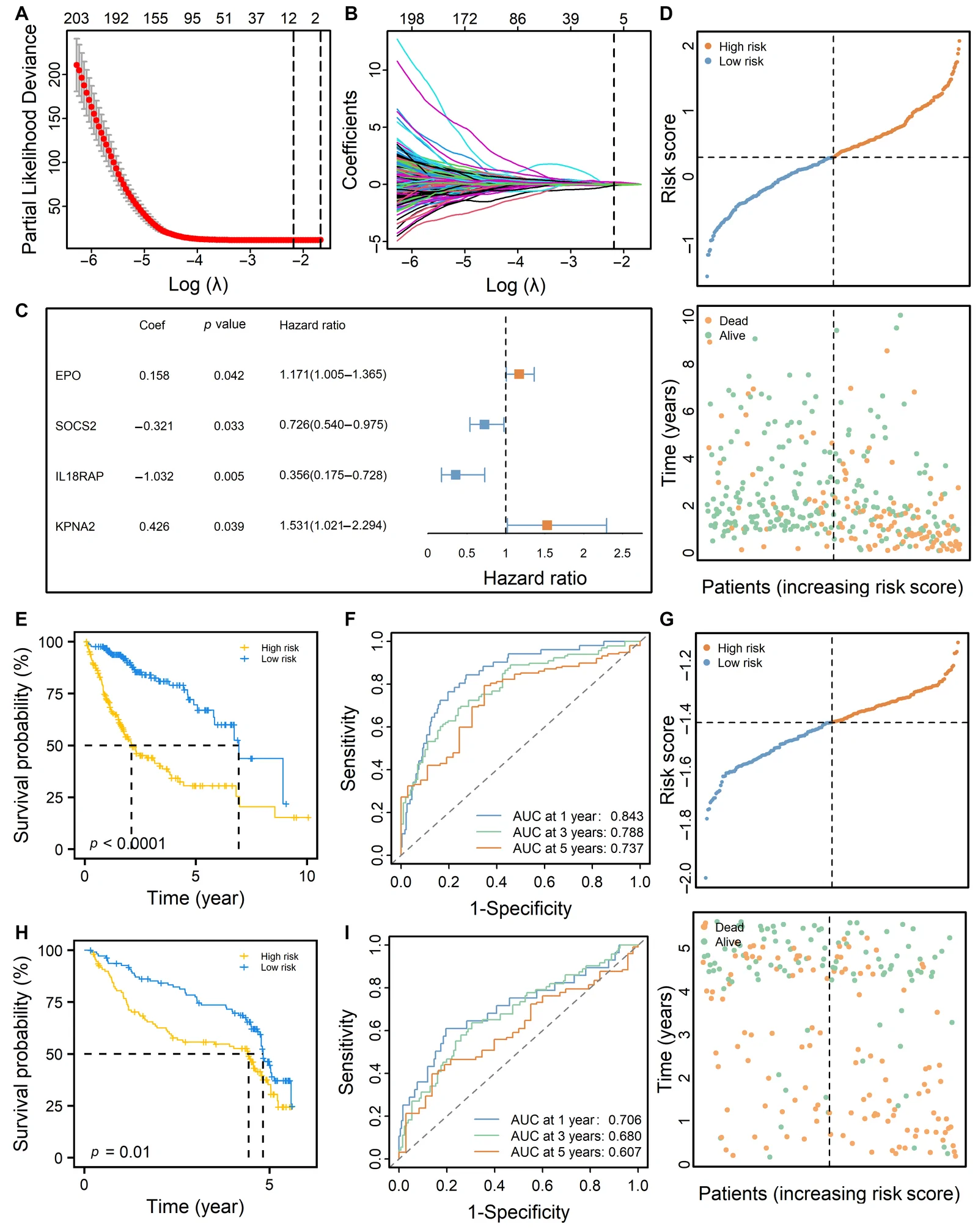
Prognostic risk model construction and verification. (**A**) The curve of cross-validation bias in LASSO regression. The vertical dashed line indicates the optimal value of log(λ) for model regularization. (**B**) The trajectory of the feature regression coefficients in LASSO analysis. The vertical dashed line indicates the optimal value of log(λ) for model regularization. (**C**) The results of multivariate Cox regression. The vertical dashed line indicates the hazard ratio of 1. (**D**) Distribution of risk scores and survival status among HCC patients grouped by risk in the TCGA-LIHC cohort. The dotted line indicates the median risk score. (**E**) KM survival distribution curves in the TCGA-LIHC dataset. The horizontal dashed line indicates the 50% survival probability. (**F**) In the TCGA-LIHC cohort, prognostic ROC curves of 1-, 3-, and 5-year OS. The diagonal dotted line is the reference line for random predictions. (**G**) The GSE14520 dataset was used to analyze the survival status and risk score distribution of HCC patients in different risk groups. The dotted line indicates the median risk score. (**H**) KM survival curves for the GSE14520 dataset. The horizontal dashed line indicates the 50% survival probability. (**I**) In the GSE14520 dataset, prognostic ROC of 1-, 3-, and 5-year OS. The diagonal dotted line is the reference line for random predictions.

To investigate the impact of DEGs on clinical survival, we analyzed 3821 DEGs using univariate Cox regression and screened out 437 DEGs that were significantly associated (Wald test, *p* < 0.001) with survival. Subsequently, using LASSO regression analysis and 10-fold cross-validation (lambda.min=0.112), we identified 10 genes markedly linked to the prognostic outcome of HCC (Figure 2A,B). Multivariate Cox regression analysis was performed on these 10 genes, and four key genes (EPO, SOCS2, IL18RAP, and KPNA2) were revealed to be significantly associated with HCC (*p* < 0.05). Among them, EPO and KPNA2 are risk factors, while SOCS2 and IL18RAP are protective factors (Figure 2C). Ultimately, the following risk scoring model was established.

$$Riskscore=0.158\times el_{EPO}-0.321\times el_{SOCS2}-1.032\times el_{IL18RAP}+0.426\times el_{KPNA2}$$

Risk scores were calculated for each participant. Using the cutoff of 0.294, HCC samples were classified into high- and low-risk groups. In TCGA-LIHC, the high-risk group has shorter overall survival than the low-risk group (Figure 2D), and Kaplan–Meier analysis shows shorter survival in the high-risk group (Figure 2E). Time-dependent ROC analysis yields AUCs of 0.843, 0.788, and 0.737 at 1, 3, and 5 years, respectively (Figure 2F). The decline in AUC over time indicated stronger short-term than long-term discrimination. The Cox-derived score was then evaluated in the external GSE14520 cohort. The high-risk group again shows shorter survival (Figure 2G,H), with AUCs of 0.706, 0.680, and 0.607 at 1, 3, and 5 years, respectively (Figure 2I). Together, these results support the reproducibility of the association between the four-gene score and survival across the two cohorts. Further prospective evaluation is required before any clinical application of this score can be considered.

### 2.3. Validation of the Expression of Four Key Genes in Different Omics Data

**Figure 3:**
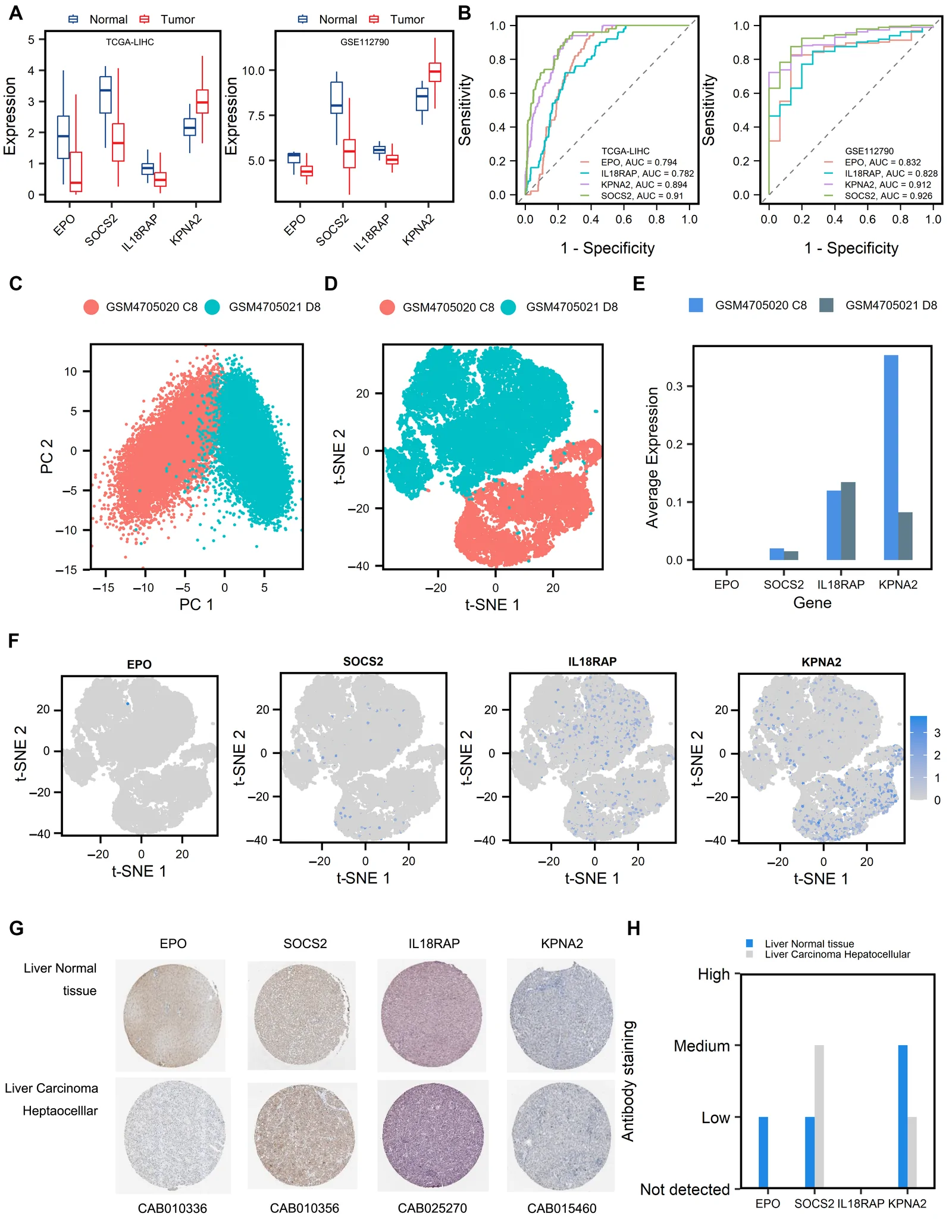
Cross-platform expression profiles of four key genes in HCC and normal liver tissues. (**A**) Differential expression of the four key genes between HCC and normal tissues in the TCGA-LIHC and GSE112790 datasets. (**B**) Receiver operating characteristic (ROC) curves for distinguishing HCC from normal tissues based on the expression of the four key genes in the TCGA-LIHC and GSE112790 datasets. The dashed line represents the reference line for random predictions (AUC=0.5). (**C**) Principal component analysis (PCA) of single-cell gene-expression profiles. (**D**) t-distributed stochastic neighbor embedding (t-SNE) of single-cell gene-expression profiles. (**E**) Mean expression levels of the four key genes in single cells. (**F**) t-SNE feature plots showing the expression of the four key genes in single cells. (**G**) Representative immunohistochemical staining of the key genes in HCC and normal tissues. (**H**) Immunohistochemical staining results for the key genes. The single-cell data comprised one HCC sample (GSM4705021, D8) and one normal-liver sample (GSM4705020, C8).

To investigate the expression variations of four key genes (EPO, SOCS2, IL18RAP, and KPNA2) in different samples, we compared their expression statuses in HCC and normal liver tissues from GSE112790, GSE14520, GSE121248, and TCGA-LIHC datasets (Figure 3A and Figure S1A). Our findings demonstrate that the expression patterns of key genes are generally consistent across different datasets. For example, EPO, SOCS2, and IL18RAP are mostly down-regulated in HCC, while KPNA2 is up-regulated in HCC. This is consistent with the fact that SOCS2 and IL18RAP are protective factors, while KPNA2 is a risk factor in the risk scoring model. Furthermore, to analyze the diagnostic performance of key gene expression in HCC, we used the expression data of four key genes from these four datasets as features and performed predictive analysis on HCC and normal tissues based on the LR algorithm. The results show that the predicted AUC values by the four key genes in the TCGA-LIHC and GSE112790 datasets are all higher than 0.78 (Figure 3B and Figure S1B), suggesting that these key genes play a beneficial role in differentiating HCC from normal tissues.

In the GSE155481 single-cell dataset, PCA and t-SNE reveal heterogeneous cellular expression profiles, and the four genes show distinct expression patterns between the HCC and normal-liver samples (Figure 3C–F). SOCS2 shows lower expression in the HCC sample than in the normal-liver sample, consistent with its expression pattern in the TCGA-LIHC, GSE121248, GSE112790, and GSE14520 datasets. Because this dataset contains only one HCC sample and one normal liver sample, the single-cell findings provide sample-level cellular context but cannot separate disease status from donor or batch effects. Therefore, these observations are exploratory and require further confirmation in multi-donor datasets.

To investigate changes in protein expression of key genes, we analyzed their immunohistochemical results in HCC and normal tissues. EPO shows stronger staining in normal tissue than in HCC tissue (Figure 3G,H), consistent with lower EPO expression in HCC. This protein-level observation provides additional context for the transcriptomic finding. Across bulk transcriptomic, single-cell, and protein-level resources, EPO, SOCS2, IL18RAP, and KPNA2 show broadly concordant, although not identical, expression patterns. These observations are consistent with their selection as prognosis-associated genes and provide biological context for the risk score. Differences in cohort origin, assay platform, and the limited single-cell design mean that these datasets should be regarded as complementary evidence rather than independent functional validation.

### 2.4. Survival Correlation Between Risk Score and Clinical Characteristics

**Figure 4:**
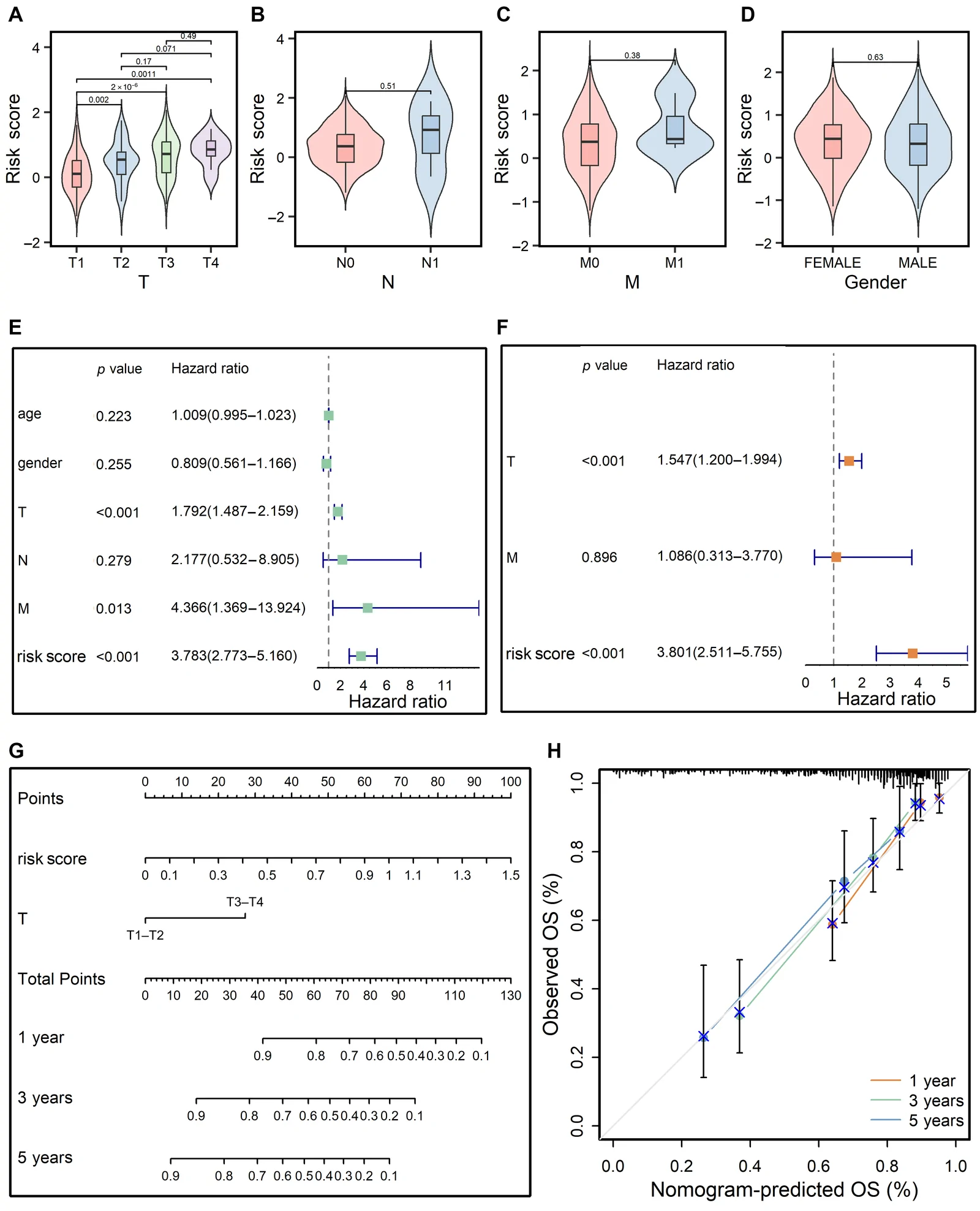
Prognostic assessment of risk score and clinical characteristics. (**A**) Risk scores among different T stages. (**B**) Risk scores among different N stages. (**C**) Risk scores among different M stages. (**D**) Risk scores among different genders. (**E**) Univariate Cox analysis on clinical indicators and risk signature. (**F**) Multivariate Cox analysis on clinical indicators and risk signature. (**G**) Nomograph based on the risk score and T stages. (**H**) The calibration curves for 1-, 3-, and 5-year OS.

To identify the correlation between risk score and multiple clinical features (T, N, M, gender, and age), we analyzed the differences between subgroups categorized by these clinical features. There is a significant difference in risk level between T1 patients and other patients with higher T stage, and the risk scores increase with increasing stage T (Figure 4A). The overall trend of risk scores across N, M, and gender subgroups indicates that higher N and M stages are associated with higher risk scores (Figure 4B–D).

Univariate Cox analysis shows that T stage (*p* < 0.001), M stage (*p* = 0.013), and the risk score (*p* < 0.001) are associated with overall survival in the TCGA-LIHC cohort (Figure 4E). In multivariate Cox analysis, the risk score (*p* < 0.001) and T stage (*p* < 0.001) remain associated with overall survival after adjustment for the included clinical variables (Figure 4F). T stage and the individualized risk score were then integrated into a nomogram for long-term survival estimation (Figure 4G). The calibration curves show agreement between nomogram-predicted and observed survival (Figure 4H). These results indicate that the risk score provides prognostic information in the analyzed cohort and may complement conventional staging for risk stratification. Its incremental value over established clinical models requires validation in independent and prospective cohorts.

### 2.5. Construction and Evaluation of Prognostic Survival Machine Learning Models Based on Key Genes

**Figure 5:**
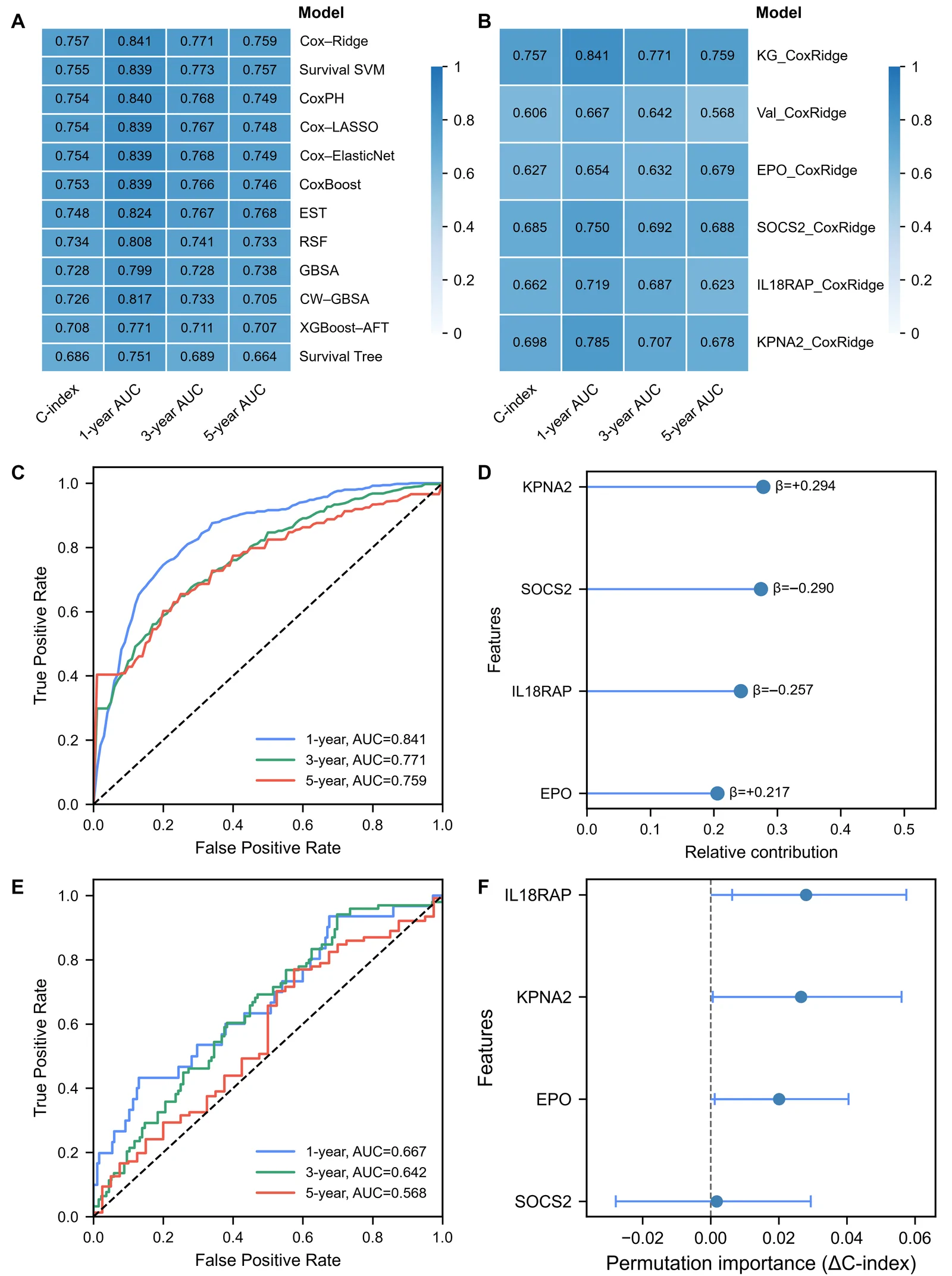
Survival machine-learning analysis based on the expression of EPO, SOCS2, IL18RAP, and KPNA2. (**A**) Performance comparison of 12 survival machine learning algorithms in TCGA-LIHC using the C-index and time-dependent AUCs at 1, 3, and 5 years. (**B**) Comparison of the four-gene Cox–Ridge model (KG_CoxRidge) in TCGA-LIHC, its evaluation in GSE14520 (Val_CoxRidge), and Cox–Ridge models based on each individual gene. (**C**) Time-dependent ROC curves for the four-gene Cox–Ridge model in TCGA-LIHC. The diagonal dotted line represents a reference line for random predictions. (**D**) Cox–Ridge coefficients of the four gene-expression predictors in TCGA-LIHC. (**E**) Time-dependent ROC curves for the four-gene Cox–Ridge model in GSE14520. The diagonal dotted line represents a reference line for random predictions. (**F**) Permutation importance of the four genes in GSE14520, expressed as the mean change in C-index after permutation. The vertical dashed line indicates a zero value for permutation importance.

To predict overall survival while retaining right-censoring information, 12 survival machine-learning algorithms were evaluated in the TCGA-LIHC cohort using the raw expression values of EPO, SOCS2, IL18RAP, and KPNA2 as predictors. The Cox-derived risk score was not included as a machine-learning input because it is a linear combination of the same four gene-expression variables. Model performance was assessed using the concordance index (C-index) and time-dependent area under the curve (AUC) values at 1, 3, and 5 years. Cox–Ridge achieved the highest C-index of 0.757, followed closely by Survival SVM with a C-index of 0.755 (Figure 5A). Cox–Ridge was therefore selected for subsequent analyses. We next compared the four-key-gene Cox–Ridge model (KG_CoxRidge), with Cox–Ridge models based on each individual gene (Figure 5B). In TCGA-LIHC, KG_CoxRidge achieved a C-index of 0.757 and time-dependent AUCs of 0.841, 0.771, and 0.759 at 1, 3, and 5 years, respectively (Figure 5C). These values are higher than those obtained from the individual-gene models. When evaluated in the independent GSE14520 cohort, the four-gene Cox–Ridge model (Val_CoxRidge) achieved a C-index of 0.606 and AUCs of 0.667, 0.642, and 0.568 at 1, 3, and 5 years, respectively (Figure 5B,E). The lower values in GSE14520 indicated attenuated discrimination in the independent cohort. In the Cox–Ridge model, positive coefficients were obtained for EPO (β=0.217) and KPNA2 (β=0.294), whereas SOCS2 (β=−0.290) and IL18RAP (β=−0.257) had negative coefficients (Figure 5D). In GSE14520, IL18RAP and KPNA2 showed the largest mean permutation-importance estimates, followed by EPO, whereas SOCS2 showed the smallest mean estimate (Figure 5F). Given the uncertainty around these estimates, their relative ranking should be interpreted with caution. The Cox–Ridge coefficients and permutation-importance estimates describe model-level predictive contributions and do not establish independent biological effects.

### 2.6. Analysis of the Tumor Immune Microenvironment

**Figure 6:**
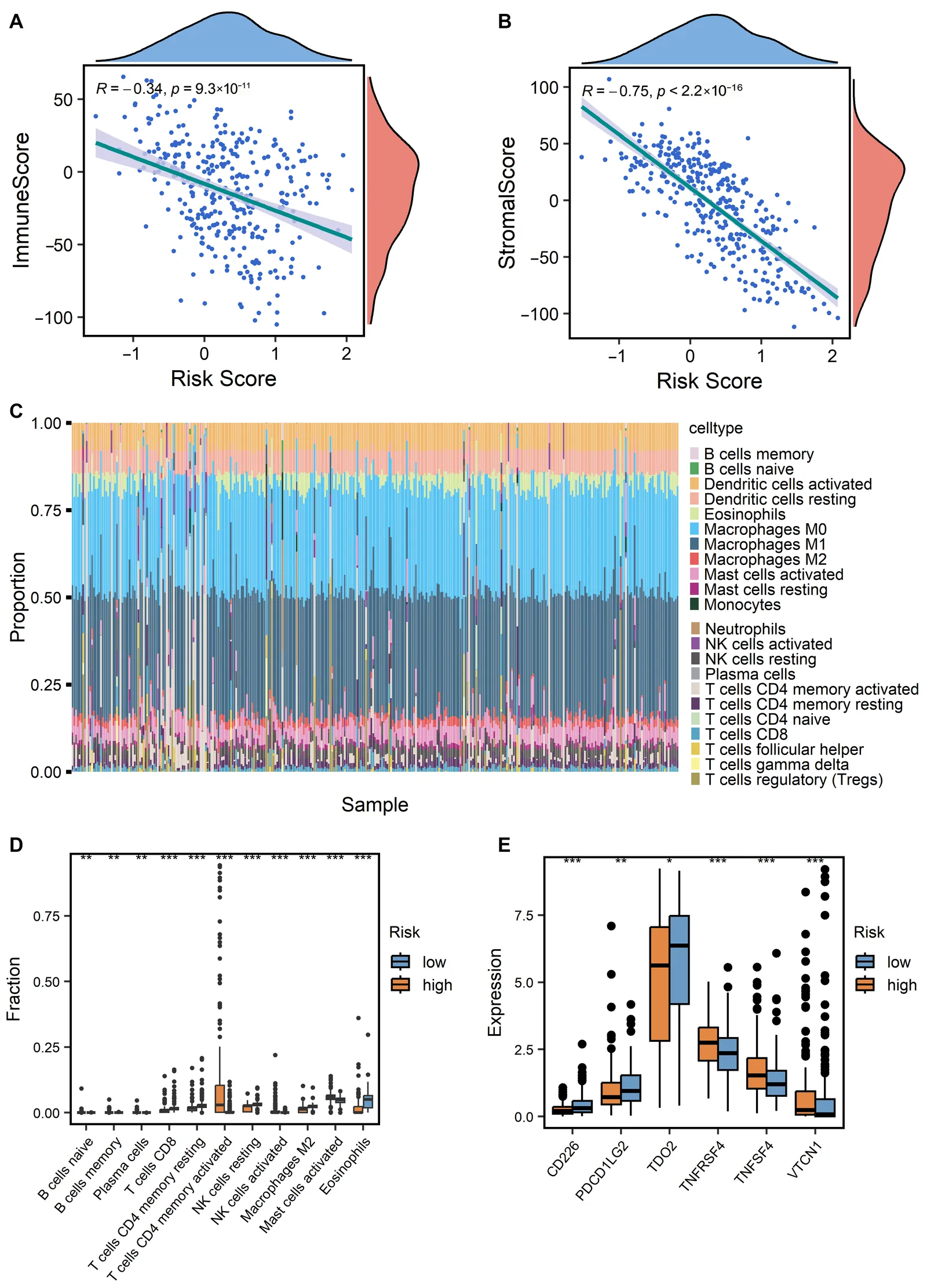
Associations of the risk score with the estimated tumor immune microenvironment in HCC. (**A**) Correlation between the risk score and the ESTIMATE-derived immune score. (**B**) Correlation between the risk score and the ESTIMATE-derived stromal score. (**C**) CIBERSORT-estimated relative abundance of 22 immune-cell populations in HCC samples. (**D**) Differences in the estimated relative abundance of immune-cell populations between high- and low-risk groups. (**E**) Differential expression of immune-checkpoint genes between high- and low-risk groups (* means adjust *p* < 0.05, ** means adjust *p* < 0.01, and *** means adjust *p* < 0.001).

To explore the immune microenvironment of HCC, we assessed correlations between the risk score and ESTIMATE-derived immune and stromal scores. Immune and stromal scores are negatively correlated with the risk score (R=−0.34 and R=−0.75, respectively) (Figure 6A,B), indicating that higher risk scores are associated with lower ESTIMATE-derived immune and stromal scores. Because the ESTIMATE immune score is a composite estimate of immune-cell content in bulk transcriptomic data rather than a direct measure of inflammatory signaling, cytokine activity, or the functional state of specific immune-cell populations, this modest correlation does not quantify the overall contribution of inflammation to hepatocarcinogenesis. The differing correlation strengths indicate that the risk score is associated differently with the estimated immune and stromal components, rather than serving as a direct surrogate for either score.

To characterize the inferred immune-cell composition, we assessed the estimated relative abundance of immune-cell populations. Macrophages M1 and M0 are among the more abundant inferred populations (Table S1 and Figure 6C). Macrophage states have previously been implicated in HCC biology [39]. We then compared estimated immune-cell abundances between the two risk groups. The high-risk group shows higher estimated proportions of B cells naive, plasma cells, Mast cells activated, T cells CD4 memory activated, NK cells activated, and B cells memory (adjusted *p* < 0.01). In contrast, eosinophils, T cells CD4 memory resting, Macrophages M2, T cells CD8, and NK cells resting show higher estimated proportions in the low-risk group (adjusted *p* < 0.01) (Figure 6D). The simultaneous elevation of activated lymphocyte populations and mast cells in high-risk tumors indicates heterogeneity within the inferred immune microenvironment. These inferred abundance estimates do not distinguish effective antitumor activation from dysfunctional or immunoregulatory states.

To assess immune-checkpoint-gene expression across risk groups, we analyzed 79 immune-checkpoint genes and adjusted the corresponding *p* values using the Benjamini–Hochberg procedure. Six genes differed significantly between the two groups after correction. TNFRSF4, TNFSF4, and VTCN1 are more highly expressed in the high-risk group (adjusted *p* < 0.05), whereas CD226, PDCD1LG2, and TDO2 are more highly expressed in the low-risk group (adjusted *p* < 0.05) (Figure 6E). TDO2 has been reported to restrain HCC-cell proliferation by upregulating p21 and p27, whereas low TDO2 expression has been associated with poor prognosis and adverse clinical outcomes [40]. The higher TDO2 expression observed in the low-risk subgroup may therefore be consistent with an antiproliferative molecular context. Together, these analyses show risk-group-associated differences in estimated immune-cell composition and immune-checkpoint expression.

### 2.7. Drug Prediction of Key Genes

**Figure 7:**
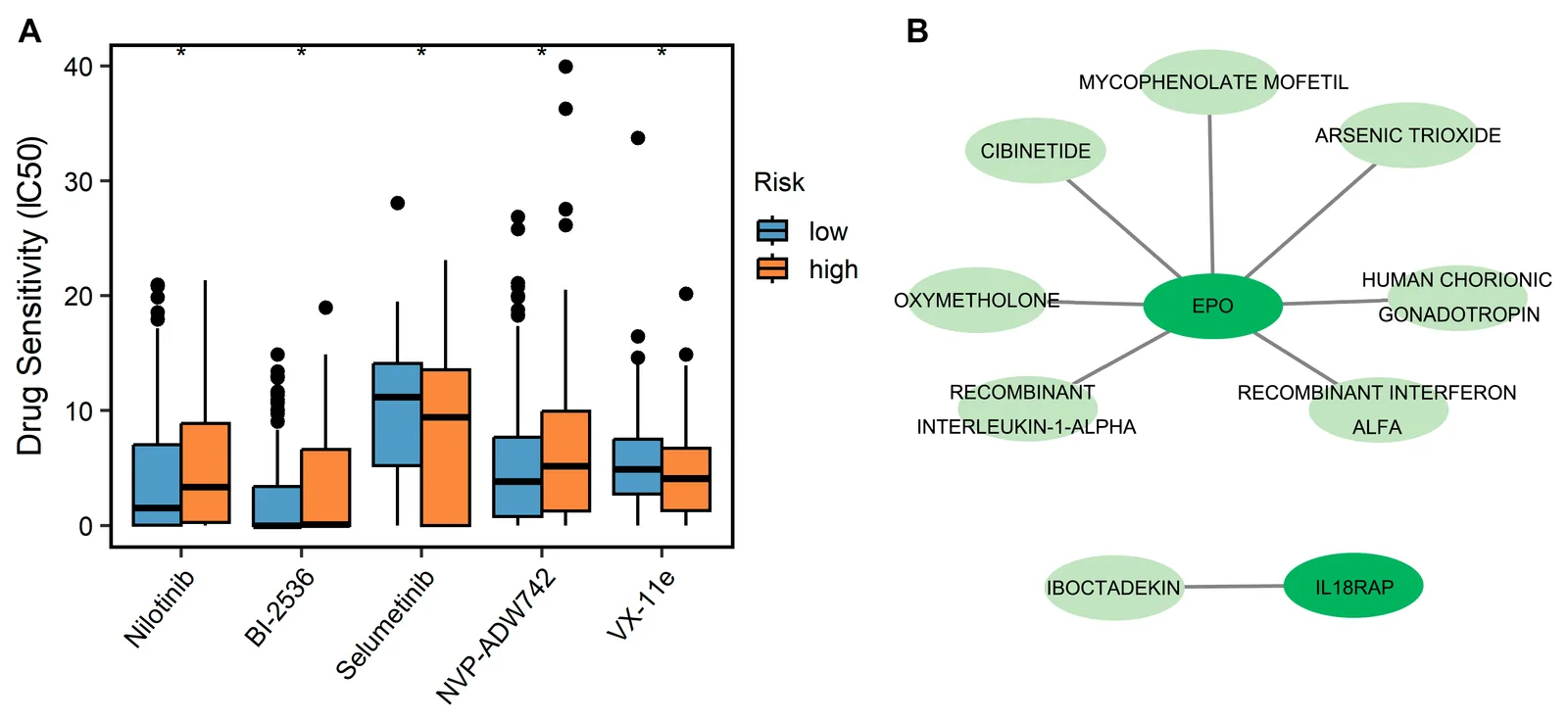
Computational drug-response predictions and database-derived gene-drug interactions. (**A**) oncoPredict/GDSC2-predicted half-maximal inhibitory concentration (IC50) values in high- and low-risk groups (* *p* < 0.05). (**B**) DGIdb-derived interactions between key genes and candidate compounds. Dark green represents key genes, while light green corresponds to candidate compounds.

To explore potential compounds associated with risk stratification, drug-response predictions were generated using oncoPredict based on the GDSC2 cell-line resource. Among the 198 screened compounds, five show nominal between-group differences in predicted IC50 values under the original unadjusted Wilcoxon screening criterion (*p* < 0.05) (Figure 7A). Nilotinib, BI-2536, and NVP-ADW742 show lower predicted IC50 values in the low-risk group, whereas Selumetinib and VX-11e show lower predicted IC50 values in the high-risk group. However, none of the 198 compounds remained significant after Benjamini–Hochberg correction (Table S2). The observed differences in predicted IC50 values are modest. Therefore, these five compounds are retained as nominal computational signals for transparency and hypothesis generation only, rather than as evidence of risk-specific therapeutic efficacy or validated therapeutic candidates.

In addition, the DGIdb database identified eight database-curated compounds associated with EPO and IL18RAP: RECOMBINANT INTERLEUKIN-1-ALPHA, OXYMETHOLONE, CIBINETIDE, HUMAN CHORIONIC GONADOTROPIN, RECOMBINANT INTERFERON ALFA, MYCOPHENOLATE MOFETIL, ARSENIC TRIOXIDE, and IBOCTADEKIN. A gene-drug interaction network was constructed using these interactions (Figure 7B). This network provides hypothesis-generating links for subsequent experimental evaluation in HCC.

## 3. Discussion

This study identified four genes associated with HCC prognosis and evaluated the censoring-aware Cox–Ridge model in TCGA-LIHC and GSE14520. The model showed internal discrimination in TCGA-LIHC, but more modest external performance in GSE14520. Cross-platform expression profiling, immune, and drug-response analyses provided complementary context. However, the retrospective design supports prognostic associations rather than causal mechanisms or clinical utility. Therefore, further prospective cohort and experimental studies are needed before these results can be applied clinically or in treatment.

Regarding the role of the four key genes involved in this study in HCC, several studies have revealed that EPO promotes the growth of HCC cells under hypoxic conditions [41]. Further, hCAR-mediated repression of EPO-EPOR signaling can suppress proliferation in human hepatoma cells, whereas recombinant EPO can partially restore HepG2 cell viability [42]. Moreover, it has recently been reported that tumor-derived EPO acts as an immunosuppressive switch in cancer immunity [43]. These are consistent with our findings that EPO is a risk factor and may contribute to poor prognosis in HCC. KPNA2 is involved in immune response, viral infection, and apoptosis, thereby promoting the development of HCC [44,45,46,47]. Increased expression of KPNA2 can promote the development of HCC [48]. Decreased expression of KPNA2 can effectively inhibit the activity of renal cell carcinoma [49]. Our findings indicate that KPNA2 is a marker of poor prognosis in HCC. Existing research has indicated the essential role of SOCS2 in regulating the proliferation ability, differentiation, and apoptosis of different types of tumor cells [50]. Additionally, studies have demonstrated that down-regulation of miR-196a/196b can inhibit the progression of HCC by regulating SOCS2 [51]. The evidence supports our finding that SOCS2 is a protective gene and has an inhibitory effect on HCC. According to research reports, IL18RAP plays a protective role in HCC [52]. Furthermore, since HCC is associated with chronic hepatitis, IL18RAP also plays an important regulatory function in the HCC microenvironment [53]. These previous studies are consistent with our research results, further confirming the reliability of our research and deepening our understanding of HCC pathogenesis.

Published HCC prognostic signatures differ substantially in gene number, cohort composition, endpoints, preprocessing procedures, and modeling strategies [35]. Liu et al. have developed a six-gene prognostic tool and reported 1-, 3-, and 5-year AUCs of 0.832, 0.850, and 0.768, respectively [54]. Shen et al. have compared Cox regression, GBM, random survival forest, survival tree, and DeepSurv in a distinct HCC setting [36]. Such results illustrate the potential utility of transcriptomic prognostic models, but performance values from non-harmonized cohorts and endpoints cannot establish superiority. The compact four-gene score should therefore be regarded as complementary to published signatures. Future head-to-head evaluation in shared cohorts, including feature selection and model fitting within a common validation design, will be required to define its relative performance.

Mounting evidence indicates that the microenvironment is crucial for the progression and development of HCC [55]. Correlation testing has revealed opposite relevance of the key gene risk score to stromal and immune scores in HCC, indicating that the risk score is associated with the estimated stromal and immune components of the tumor microenvironment. Specifically, patients with high-risk HCC show a significant increase in the enrichment fractions of Mast cells activated, NK cells activated, and T cells CD4 memory activated (*p* < 0.001). Studies have shown that CD4 T cells have a specific killing effect on HCC cells [56]. NK cells can mediate the death of HCC cells [57]. These observations are consistent with the inferred immune-cell patterns and provide hypotheses for further investigation. In addition, the microenvironment of HCC is usually in a state of chronic inflammation. Accumulating evidence has demonstrated that mast cells in the HCC microenvironment promote the proliferation of HCC cells by secreting inflammatory mediators [58]. This finding was consistent with the higher inferred abundance of activated mast cells in the high-risk group. However, because these estimates were derived from bulk-transcriptomic deconvolution, they cannot establish mast-cell function, cellular mechanisms, or immune tolerance. Furthermore, our study also identified that the expression of TNFRSF4, TNFSF4, and VTCN1 is significantly increased in high-risk individuals. Previous studies have demonstrated a significant upregulation of TNFRSF4 in HCC [59]. In esophageal squamous cell carcinoma, TNFSF4-TNFRSF4 is a potential target for immunotherapy [60]. The activation, proliferation, and secretion of pro-inflammatory mediators in effector T cells can be inhibited by VTCN1, leading to the construction of a tumor immune suppression milieu [61]. These studies provide biological context for the observed checkpoint-gene associations. However, these analyses have not identified therapeutic targets or therapeutic responsiveness. Furthermore, how these biological processes exert regulatory effects and their detailed molecular basis in HCC malignant progression still remains ambiguous; thus, further systematic studies are urgently needed.

TDO2 is more highly expressed in the low-risk subgroup [62], despite its reported roles in tryptophan-kynurenine metabolism and immune regulation. This pattern underscores that checkpoint-gene expression may reflect tumor purity and cellular composition, in addition to tumor-cell-intrinsic programs. In this cohort, higher TDO2 expression should be interpreted as a correlate of the observed low-risk immune context rather than evidence of a protective effect. In the future, independent cohorts with matched assessments of tumor purity and immune infiltration need to be established and experimentally validated to determine whether this pattern reflects cellular composition, microenvironmental state, or tumor cell-intrinsic regulation.

However, this study has limitations. This retrospective study used public datasets with heterogeneous platforms and clinical annotations. The four-gene set was selected in the original TCGA-LIHC discovery stage; the Cox–Ridge analysis evaluated pre-defined gene characteristics rather than de novo feature selection within each resampling fold. The single-cell comparison included only one HCC and one normal liver sample, precluding separation of disease, donor, and batch effects. Drug response estimates were derived from 2D cell-line data, and none of the drug signals remained significant after correction across 198 tests. Finally, due to limitations in experimental conditions, no available laboratory platform was available for this retrospective public data study; therefore, wet experimental validation was not performed. In the future, we will need to use more reliable data and experimental validation to determine the biological characteristics and clinical applications of these features.

## 4. Materials and Methods

### 4.1. Sources of Transcriptome Data and Clinical Data

We obtained and organized TCGA-LIHC data through the GDC portal (https://portal.gdc.cancer.gov/, accessed on 22 June 2024). The dataset comprised bulk RNA-seq profiles in counts and TPM formats from 374 HCC and 50 non-tumor samples, together with clinical information from 377 HCC patients. Public GEO resources (https://www.ncbi.nlm.nih.gov/geo/, accessed on 21 November 2024) included three gene-expression datasets (GSE14520, GSE112790, and GSE121248) and one single-cell RNA-sequencing dataset (GSE155481). GSE14520 included gene-expression profiles from 225 HCC tissues and 220 normal samples, together with survival data from 242 HCC patients. GSE112790 included gene-expression profiles from 183 HCC and 15 normal tissues, whereas GSE121248 included profiles from 37 HCC and 70 normal tissues. GSE155481 provided single-cell RNA-sequencing data from one HCC sample (GSM4705021, D8) and one normal liver sample (GSM4705020, C8).

### 4.2. Processing of Gene Expression Data and Differential Expression Analysis

First, the TCGA transcriptomic matrix was organized, 369 HCC and 50 normal samples were screened from 424 samples. Second, for duplicate genes within the count matrix, only the record with the largest expression value was retained, leaving 19,937 protein-coding genes for subsequent analysis. Then, genes with the mean expression level below 1 across all samples were removed, leaving 17,490 genes. To further filter out genes with low expression levels, we standardized the gene expression data to counts per million (*CPM*) according to Equation (1), and then selected genes with *CPM* greater than one in at least two samples, resulting in 16,851 genes. Finally, we selected the same genes from the downloaded *TPM* (transcripts per million) data for subsequent analysis.

(1) $$CPM_{i}=(\alpha_{i}\times 10^{6})/\sum_{i=1}^{n}\alpha_{i}$$

where i indicates the *i*-*th* gene, $\alpha_{i}$ indicates the total reads aligned with exonic segments of the *i*-*th* gene, and n denotes the total number of genes.

After the expression filtering steps described above, edgeR was used to calculate normalization factors. The filtered count matrix was then processed using the limma-voom transformation with quantile normalization. A linear model and empirical Bayes moderation were applied to compare tumor and non-tumor samples. Differentially expressed genes were defined by $\left|\log_{2}FoldChange\right|>1$ and Benjamini–Hochberg adjusted *p* < 0.05, yielding 3821 genes [63].

### 4.3. Gene Functional Annotation and Pathway Enrichment Analysis

Gene Ontology (GO) functional annotation and Kyoto Encyclopedia of Genes and Genomes (KEGG) pathway enrichment analysis were performed with clusterProfiler (v4.12.6) in R (v4.4.1) [64]. Using *p* < 0.05 and q<0.05 as thresholds, the cellular components (CC), molecular functions (MF), biological processes (BP), and pathways associated with HCC were screened.

### 4.4. Construction of the Prognosis Risk Scoring Model

Clinical information was retrieved for 377 TCGA-LIHC patients, of whom 349 with an overall survival time exceeding 30 days were retained for subsequent analysis. These data were integrated with normalized gene-expression profiles from 369 HCC samples, yielding 341 overlapping samples. LASSO regression (glmnet, v4.1-8), univariate Cox regression, and multivariate Cox regression (survival, v3.7-0) were performed [65]. Ten-fold cross-validation was used to select the LASSO regularization parameter. Based on multivariate Cox regression, a four-gene prognostic risk score was established, as presented in Equation (2). Because this score is a deterministic linear combination of the expression values of EPO, SOCS2, IL18RAP, and KPNA2, it was not included as an additional predictor in the survival machine-learning analyses. External evaluation in GSE14520 was performed by applying the coefficients derived from TCGA-LIHC without refitting the Cox risk-score model.

(2) $$Riskscore=\sum_{i=1}^{n}coef\left({\mathrm{gene}}_{i}\right)\times el\left({\mathrm{gene}}_{i}\right)$$

where n denotes the total number of genes, i is the gene, $coef\left({\mathrm{gene}}_{i}\right)$ denotes the regression coefficient of gene i, $el\left(gene_{i}\right)$ denotes the expression level for the gene i.

### 4.5. Survival Analysis

The prognostic performance of the Cox-derived risk score was assessed using Kaplan–Meier survival analysis (survminer, v0.5.0), nomogram analysis (rms, v6.8-2), time-dependent ROC curves (timeROC, v0.4), and calibration curves. Patients were classified into high- and low-risk groups according to the median risk score. Clinical variables, including T stage, N stage, M stage, sex, and age, were included in the nomogram analysis. The Cox, Kaplan–Meier, time-dependent ROC, and calibration analyses used the observed overall-survival time and censoring indicator.

### 4.6. Single-Cell Gene Expression Analysis

To investigate gene expression in single cells of immune phenotypes from HCC and normal liver tissues, we first performed quality control and filtering (Seurat, v5.1.0) on the GSE155481 single-cell data [66]. Cells with 200 to 2500 detected genes were retained, and genes expressed in at least two cells were included. Cells with more than 5% mitochondrial gene content were excluded. The top 2000 highly variable genes were selected for subsequent analysis. The cellular transcriptional landscape was subsequently visualized using principal component analysis (PCA) and t-distributed stochastic neighbor embedding (t-SNE). Given the single-sample design of the dataset, the single-cell analysis was limited to sample-level visualization.

### 4.7. Protein Differential Analysis

To evaluate differential protein levels of key genes across normal and HCC tissues. We obtained expression data (immunohistochemistry, IHC) of relevant proteins from the Human Protein Atlas (HPA) database (https://www.proteinatlas.org/, accessed on 22 November 2024) and analyzed the protein expression patterns of key genes in different samples.

### 4.8. Construction and External Evaluation of Prognostic Survival Machine-Learning Models

Overall survival time and event status were used as the outcomes for all survival machine-learning analyses. The analysis included 341 TCGA-LIHC patients with complete four-gene expression and survival information. EPO, SOCS2, IL18RAP, and KPNA2 were used as predictors. The previously established Cox-derived risk score was excluded because it was calculated from these four genes. To reduce platform-related differences between TCGA RNA-seq data and the external microarray data, each gene was independently transformed into a percentile rank ranging from 0 to 1 within each cohort. This transformation used expression data only and did not use survival outcomes.

Twelve survival machine learning algorithms were evaluated, including Cox proportional hazards regression (CoxPH), CoxBoost, Cox-LASSO, Cox–Ridge, Cox-ElasticNet, random survival forest (RSF), extremely randomized survival trees (EST), survival tree, gradient boosting survival analysis (GBSA), component-wise gradient boosting survival analysis (CW-GBSA), Survival SVM, and XGBoost accelerated failure time (XGBoost-AFT). Model performance was assessed using 10 repeats of stratified five-fold cross-validation with identical fold assignments across models. When hyperparameter tuning was required, model parameters were determined using training data only. Harrell’s concordance index (C-index) was used as the primary evaluation metric, together with time-dependent AUCs at 1, 3, and 5 years. Time-dependent ROC curves at 1, 3, and 5 years were generated from held-out TCGA predictions.

The final Cox–Ridge model was fitted using all eligible TCGA-LIHC patients. The relative contribution of each gene was calculated from the absolute value of its Cox–Ridge coefficient:

(3) $$\mathrm{Relative}\;{\mathrm{contribution}}_{i}=\frac{\left|\beta_{i}\right|}{\sum_{j=1}^{4}\left|\beta_{j}\right|}$$

where $\beta_{i}$ represents the Cox–Ridge coefficient for gene i. The absolute coefficient magnitude represents the relative contribution of each gene, whereas the original coefficient sign indicates its direction within the model. These coefficient-based contributions describe model-level predictive contributions.

For external evaluation, the fitted TCGA Cox–Ridge model and its coefficients were frozen and directly applied to 221 GSE14520 patients with complete four-gene expression and survival information. No coefficient re-estimation, outcome-based parameter selection, or model refitting was performed. Survival time in GSE14520 was converted from months to years. The four gene-expression features were transformed into percentile ranks within the external cohort using the prespecified predictor-only procedure. External performance was evaluated using the C-index and time-dependent AUCs at 1-, 3-, and 5-years.

Permutation importance was assessed in GSE14520 to examine the contribution of each gene to the predictive performance of the frozen model. Each percentile-rank-transformed gene was independently permuted 500 times, while the other three features remained unchanged. For permutation b, the decrease in C-index was calculated as:

(4) $$\Delta C_{i}^{\left(b\right)}=C_{\mathrm{baseline}}-C_{i,\mathrm{permuted}}^{\left(b\right)}$$

The mean ΔC-index and the empirical 95% permutation interval, defined by the 2.5th and 97.5th percentiles of the permutation distribution, were reported. Positive values indicated reduced predictive performance after permutation, whereas values close to or below zero indicated limited or unstable predictive contribution in the external cohort.

### 4.9. Immunological Analysis of the Tumor Microenvironment

To investigate immune-related characteristics of the HCC tumor microenvironment, TCGA-LIHC gene-expression data were used to estimate stromal and immune scores with ESTIMATE (v1.0.13) [67]. CIBERSORT (v0.1.0) was then applied to infer the relative abundance of 22 immune-cell populations. Correlations between stromal or immune scores and the risk score were assessed, and differences in estimated immune-cell abundance and immune-checkpoint gene expression were compared between the high- and low-risk groups [68]. For the immune-checkpoint analysis, *p* values for the 79 immune checkpoint genes were adjusted using the Benjamini–Hochberg procedure, and the adjusted *p* < 0.05 was considered statistically significant. These bulk-transcriptomic deconvolution estimates were interpreted as associative findings and do not establish cellular mechanisms, changes in HCC risk, or therapeutic responsiveness.

### 4.10. Drug Sensitivity Analysis

To screen candidate drugs, drug-sensitivity prediction was performed using oncoPredict (v1.2) with GDSC2 cell line data [69,70,71]. GDSC2 data were obtained from the Genomics of Drug Sensitivity in Cancer database (https://www.cancerrxgene.org/, accessed on 7 December 2024) and integrated with the expression profiles of DEGs to estimate predicted IC50 values for 198 drugs in TCGA-LIHC samples. The original screen compared predicted responses between risk groups using Wilcoxon tests and nominated compounds at an unadjusted *p* < 0.05. Drug–gene interaction information was obtained from DGIdb (https://dgidb.org/, accessed on 12 December 2024), and Cytoscape (v3.7.1) was used to construct a drug–gene interaction network. These predictions were derived from 2D cell-line data. They were used to generate hypotheses for experimental prioritization rather than evidence of clinical drug sensitivity, tumor-microenvironment effects, or in vivo efficacy. The 198 Wilcoxon *p* values were adjusted using the Benjamini–Hochberg procedure, and an FDR-adjusted q<0.05 was considered statistically significant.

### 4.11. Statistical Analysis

A series of bioinformatics analyses and computational procedures were carried out with R (v4.4.1) and Python (v3.13.0).

## 5. Conclusions

In conclusion, we identified four genes associated with HCC prognosis and evaluated a Cox-derived score across independent cohorts. A censoring-aware Cox–Ridge model based on the four genes showed internal discrimination in TCGA-LIHC and more modest external performance in GSE14520. Cross-platform expression, immune, and drug-response analyses provided complementary and hypothesis-generating context. The computational drug findings do not establish therapeutic efficacy. Prospective cohorts and experimental studies are required before clinical or therapeutic application.

## Data Availability

We have shared the link to our data in the Manuscript. We have used the gene expression data and clinical information data from the TCGA database (https://portal.gdc.cancer.gov/, accessed on 22 June 2024), the gene expression data and single-cell RNA sequencing data from the GEO database (https://www.ncbi.nlm.nih.gov/geo/, accessed on 21 November 2024), expression data of relevant proteins from the Human Protein Atlas database (https://www.proteinatlas.org/, accessed on 22 November 2024), the Genomics of Drug Sensitivity in Cancer 2 dataset from the GDSC database (https://www.cancerrxgene.org/, accessed on 7 December 2024), drug–gene interaction data from the DGIdb database (https://dgidb.org/, accessed on 12 December 2024). These data are publicly available.

## Supplementary Materials

The following supporting information can be downloaded at: https://www.mdpi.com/article/10.3390/ijms27177535/s1.

## Abbreviations

The following abbreviations are used in this manuscript:

HCC	hepatocellular carcinoma
Cox	Cox proportional hazards model
ML	machine learning
OS	overall survival
AUC	area under the receiver operating characteristic curve
LASSO	least absolute shrinkage and selection operator
XGBoost	extreme gradient boosting
SVM	support vector machine
DEGs	differentially expressed genes
GO	Gene Ontology
KEGG	Kyoto Encyclopedia of Genes and Genomes
