# Supplementary material for: Integrated Transcriptomic Analyses Identify Four Prognosis-Associated Genes in Hepatocellular Carcinoma
Source: Int J Mol Sci. 2026 Aug 23;27(17):7535. doi: 10.3390/ijms27177535 (PMC13566057; doi:10.3390/ijms27177535)
Supplement: Supplementary file 1 [file ijms-27-07535-s001.zip › ijms-4473675-supplementary.pdf]

Supplementary material for

Integrated Transcriptomic Analyses Identify Four Prognosis-Associated Genes in Hepatocellular Carcinoma

Figure S1. Differences in the expression of key genes and their predictive value for HCC and normal tissues. (A) Differences in the expression of key genes between HCC and normal tissues in the GSE14520 and GSE121248 datasets. (B) ROC curves for predicting HCC and normal tissues based on the expression of key genes in the GSE14520 and GSE121248 datasets.

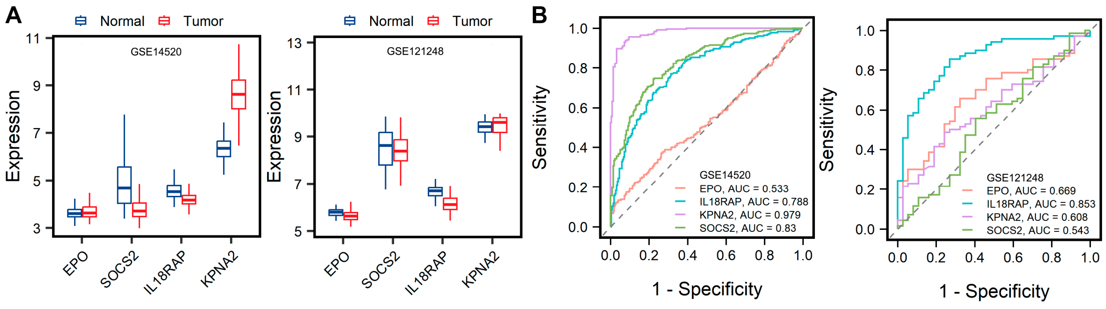

Table S1. Infiltration abundance of 22 immune cell types.

Cell types	Proportion
Macrophages M1	0.311
Macrophages M0	0.278
Dendritic cells activated	0.071
T cells CD4 memory activated	0.067
Dendritic cells resting	0.059
Mast cells activated	0.052
Eosinophils	0.032
NK cells resting	0.026
T cells CD4 memory resting	0.025
Macrophages M2	0.018
T cells CD8	0.015
T cells regulatory (Tregs)	0.015
Mast cells resting	0.011
NK cells activated	0.006
T cells follicular helper	0.006
Neutrophils	0.003
T cells gamma delta	0.001
Monocytes	0.001
T cells CD4 naive	0.0008
B cells memory	0.0005
B cells naive	0.0005
Plasma cells	0.0004

Table S2. Predicted drug sensitivity statistics of risk subgroups.

Drug	Raw P	FDR (BH)	Test status	Skipping reason
Camptothecin	0.9907	0.9925	Tested	NA
Vinblastine	0.9890	0.9925	Tested	NA
Cisplatin	0.7605	0.9925	Tested	NA
Cytarabine	0.5888	0.9925	Tested	NA
Docetaxel	0.9833	0.9925	Tested	NA
Gefitinib	0.8557	0.9925	Tested	NA
Navitoclax	0.8120	0.9925	Tested	NA
Vorinostat	0.2382	0.9925	Tested	NA
Nilotinib	0.0396	0.9925	Tested	NA
Olaparib	0.9706	0.9925	Tested	NA
Axitinib	0.5031	0.9925	Tested	NA
AZD7762	0.8635	0.9925	Tested	NA
SB216763	0.1948	0.9925	Tested	NA
KU-55933	0.2086	0.9925	Tested	NA
Afatinib	0.8450	0.9925	Tested	NA
Staurosporine	0.2391	0.9925	Tested	NA
PLX-4720	0.9925	0.9925	Tested	NA
NU7441	0.3587	0.9925	Tested	NA
Doramapimod	0.3997	0.9925	Tested	NA
Wee1 Inhibitor	0.8242	0.9925	Tested	NA
Nutlin-3a (-)	0.5706	0.9925	Tested	NA
Mirin	0.0910	0.9925	Tested	NA
PD173074	0.1688	0.9925	Tested	NA
ZM447439	0.5607	0.9925	Tested	NA
Alisertib	0.6516	0.9925	Tested	NA
RO-3306	0.4028	0.9925	Tested	NA
MK-2206	0.3610	0.9925	Tested	NA
Palbociclib	0.9182	0.9925	Tested	NA
Dactolisib	0.9636	0.9925	Tested	NA
Pictilisib	0.8747	0.9925	Tested	NA
AZD8055	0.8652	0.9925	Tested	NA
PD0325901	0.0980	0.9925	Tested	NA
Obatoclax Mesylate	0.9129	0.9925	Tested	NA
5-Fluorouracil	0.8903	0.9925	Tested	NA
Dasatinib	0.6703	0.9925	Tested	NA
Paclitaxel	0.7700	0.9925	Tested	NA
Crizotinib	0.3214	0.9925	Tested	NA
Rapamycin	0.3875	0.9925	Tested	NA
Sorafenib	0.7865	0.9925	Tested	NA
BI-2536	0.0144	0.9925	Tested	NA
Irinotecan	0.5460	0.9925	Tested	NA
Oxaliplatin	0.6183	0.9925	Tested	NA

BMS-536924	0.5828	0.9925	Tested	NA
GSK1904529A	0.5831	0.9925	Tested	NA
Tozasertib	0.6378	0.9925	Tested	NA
PF-4708671	0.9444	0.9925	Tested	NA
PRIMA-1MET	0.5786	0.9925	Tested	NA
Erlotinib	0.4103	0.9925	Tested	NA
Niraparib	0.4499	0.9925	Tested	NA
MK-1775	0.7510	0.9925	Tested	NA
Dinaciclib	0.3771	0.9925	Tested	NA
Gemcitabine	0.0963	0.9925	Tested	NA
Bortezomib	0.6603	0.9925	Tested	NA
GSK269962A	0.4494	0.9925	Tested	NA
SB505124	0.5689	0.9925	Tested	NA
Tamoxifen	0.7638	0.9925	Tested	NA
Fulvestrant	0.2462	0.9925	Tested	NA
EPZ004777	0.2662	0.9925	Tested	NA
YK-4-279	0.7272	0.9925	Tested	NA
Daporinad	0.6761	0.9925	Tested	NA
BMS-345541	0.5950	0.9925	Tested	NA
AZ960	0.7043	0.9925	Tested	NA
Talazoparib	0.4694	0.9925	Tested	NA
XAV939	0.1234	0.9925	Tested	NA
Trametinib	0.4351	0.9925	Tested	NA
Dabrafenib	0.6182	0.9925	Tested	NA
Temozolomide	0.2594	0.9925	Tested	NA
AZD5438	0.1882	0.9925	Tested	NA
IAP_5620	0.2407	0.9925	Tested	NA
AZD2014	0.3507	0.9925	Tested	NA
AZD1208	0.8222	0.9925	Tested	NA
AZD1332	0.5010	0.9925	Tested	NA
Ruxolitinib	0.8834	0.9925	Tested	NA
Linsitinib	0.2789	0.9925	Tested	NA
Epirubicin	0.1777	0.9925	Tested	NA
Cyclophosphamide	0.8635	0.9925	Tested	NA
Pevonedistat	0.6075	0.9925	Tested	NA
Sapitinib	0.9435	0.9925	Tested	NA
Uprosertib	0.6129	0.9925	Tested	NA
LCL161	0.4159	0.9925	Tested	NA
Lapatinib	0.7067	0.9925	Tested	NA
Luminespib	0.8412	0.9925	Tested	NA
Alpelisib	0.8669	0.9925	Tested	NA
Taselisib	0.7260	0.9925	Tested	NA
EPZ5676	0.3744	0.9925	Tested	NA
SCH772984	0.3066	0.9925	Tested	NA

IWP-2	0.3768	0.9925	Tested	NA
Leflunomide	0.7992	0.9925	Tested	NA
Entinostat	0.6504	0.9925	Tested	NA
OSI-027	0.6937	0.9925	Tested	NA
LGK974	0.4267	0.9925	Tested	NA
VE-822	0.3356	0.9925	Tested	NA
WZ4003	0.0650	0.9925	Tested	NA
CZC24832	0.6687	0.9925	Tested	NA
AZD5582	0.7380	0.9925	Tested	NA
GSK2606414	0.4620	0.9925	Tested	NA
PFI3	0.2039	0.9925	Tested	NA
PCI-34051	0.7992	0.9925	Tested	NA
Wnt-C59	0.2828	0.9925	Tested	NA
I-BET-762	0.4428	0.9925	Tested	NA
RVX-208	0.5380	0.9925	Tested	NA
OTX015	0.3257	0.9925	Tested	NA
GSK343	0.5366	0.9925	Tested	NA
ML323	0.9540	0.9925	Tested	NA
Entospletinib	0.6856	0.9925	Tested	NA
PRT062607	0.3738	0.9925	Tested	NA
Ribociclib	0.5409	0.9925	Tested	NA
AGI-6780	0.2228	0.9925	Tested	NA
Picolinici-acid	0.1447	0.9925	Tested	NA
AZD5153	0.1719	0.9925	Tested	NA
CDK9_5576	0.6481	0.9925	Tested	NA
CDK9_5038	0.6121	0.9925	Tested	NA
Eg5_9814	0.9590	0.9925	Tested	NA
ERK_2440	0.1398	0.9925	Tested	NA
ERK_6604	0.0589	0.9925	Tested	NA
IRAK4_4710	0.8381	0.9925	Tested	NA
JAK1_8709	0.6106	0.9925	Tested	NA
AZD5991	0.1690	0.9925	Tested	NA
PAK_5339	0.3230	0.9925	Tested	NA
TAF1_5496	0.3389	0.9925	Tested	NA
ULK1_4989	0.9086	0.9925	Tested	NA
VSP34_8731	0.3274	0.9925	Tested	NA
Selumetinib	0.0398	0.9925	Tested	NA
IGF1R_3801	0.9794	0.9925	Tested	NA
JAK_8517	0.9260	0.9925	Tested	NA
AZD4547	0.1239	0.9925	Tested	NA
Ibrutinib	0.9557	0.9925	Tested	NA
Zoledronate	0.6687	0.9925	Tested	NA
Acetalax	0.4039	0.9925	Tested	NA
Oxaliplatin	0.6183	0.9925	Tested	NA

Carmustine	0.8299	0.9925	Tested	NA
Topotecan	0.7546	0.9925	Tested	NA
Teniposide	0.4222	0.9925	Tested	NA
Mitoxantrone	0.9549	0.9925	Tested	NA
Dactinomycin	0.9130	0.9925	Tested	NA
Fludarabine	0.6437	0.9925	Tested	NA
Nelarabine	0.7921	0.9925	Tested	NA
Fulvestrant	0.2462	0.9925	Tested	NA
Vincristine	0.7681	0.9925	Tested	NA
Docetaxel	0.9833	0.9925	Tested	NA
Podophyllotoxin bromide	0.8915	0.9925	Tested	NA
Dihydrorotenone	0.3289	0.9925	Tested	NA
Gallibiscoquinazole	0.2923	0.9925	Tested	NA
Elephantin	0.8358	0.9925	Tested	NA
Sinularin	0.4442	0.9925	Tested	NA
Sabutoclax	0.5712	0.9925	Tested	NA
LY2109761	0.5497	0.9925	Tested	NA
OF-1	0.2829	0.9925	Tested	NA
MN-64	0.3644	0.9925	Tested	NA
KRAS (G12C) Inhibitor-12	0.8117	0.9925	Tested	NA
MG-132	0.6014	0.9925	Tested	NA
BDP-00009066	0.7253	0.9925	Tested	NA
Buparlisib	0.8171	0.9925	Tested	NA
Ulixertinib	0.7388	0.9925	Tested	NA
Venetoclax	0.9256	0.9925	Tested	NA
ABT737	0.1673	0.9925	Tested	NA
Dactinomycin	0.9130	0.9925	Tested	NA
Afuresertib	0.4078	0.9925	Tested	NA
AGI-5198	0.8566	0.9925	Tested	NA
AZD3759	0.3604	0.9925	Tested	NA
AZD5363	0.2989	0.9925	Tested	NA
AZD6738	0.7796	0.9925	Tested	NA
AZD8186	0.8726	0.9925	Tested	NA
Osimertinib	0.7075	0.9925	Tested	NA
Cediranib	0.3792	0.9925	Tested	NA
Ipatasertib	0.4299	0.9925	Tested	NA
GDC0810	0.6347	0.9925	Tested	NA
GNE-317	0.9321	0.9925	Tested	NA
GSK2578215A	0.5571	0.9925	Tested	NA
I-BRD9	0.7730	0.9925	Tested	NA
Telomerase Inhibitor IX	0.2210	0.9925	Tested	NA
MIRA-1	0.2440	0.9925	Tested	NA
NVP-ADW742	0.0432	0.9925	Tested	NA
P22077	0.2241	0.9925	Tested	NA

Savolitinib	0.1939	0.9925	Tested	NA
UMI-77	0.5269	0.9925	Tested	NA
WIKI4	0.4010	0.9925	Tested	NA
Sepantronium bromide	0.8898	0.9925	Tested	NA
MIM1	0.8214	0.9925	Tested	NA
WEHI-539	0.3423	0.9925	Tested	NA
BPD-00008900	0.3225	0.9925	Tested	NA
Foretinib	0.4674	0.9925	Tested	NA
BIBR-1532	0.7663	0.9925	Tested	NA
Pyridostatin	0.8064	0.9925	Tested	NA
AMG-319	0.8462	0.9925	Tested	NA
MK-8776	0.9803	0.9925	Tested	NA
Ulixertinib	0.7388	0.9925	Tested	NA
Vinorelbine	0.6561	0.9925	Tested	NA
VX-11e	0.0456	0.9925	Tested	NA
Uprosertib	0.6129	0.9925	Tested	NA
LJI308	0.5144	0.9925	Tested	NA
AZ6102	0.8413	0.9925	Tested	NA
GSK591	0.8782	0.9925	Tested	NA
VE821	0.7529	0.9925	Tested	NA
AZD6482	0.8765	0.9925	Tested	NA
AT13148	0.7297	0.9925	Tested	NA
BMS-754807	0.6488	0.9925	Tested	NA
JQ1	0.8756	0.9925	Tested	NA
